# ShapeR: An R Package to Study Otolith Shape Variation among Fish Populations

**DOI:** 10.1371/journal.pone.0121102

**Published:** 2015-03-24

**Authors:** Lísa Anne Libungan, Snæbjörn Pálsson

**Affiliations:** Department of Life and Environmental Sciences, University of Iceland, Reykjavík, Iceland; Department of Agriculture, AUSTRALIA

## Abstract

ShapeR is an open source software package that runs on the R platform and is specifically designed to study otolith shape variation among fish populations. The package extends previously described software used for otolith shape analysis by allowing the user to automatically extract closed contour outlines from a large number of images, perform smoothing to eliminate pixel noise, choose from conducting either a Fourier or Wavelet transform to the outlines and visualize the mean shape. The output of the package are independent Fourier or Wavelet coefficients which can be directly imported into a wide range of statistical packages in R. The package might prove useful in studies of any two dimensional objects.

## Introduction

Morphometric analysis of otoliths is a well-established method to delineate fish stocks, characterize population movements and to detect the natal origin of fish. For otolith shape analysis, two main morphometric methods are used: landmark analysis [1] and outline analysis [2–5]. With outline analysis it is possible to quantify boundary shapes so that patterns of shape variation within and among groups can be evaluated based on a large number of independent variables [6]. The advantage of using such methods in population identification is that they are cost effective and only require otolith images from which outlines can be extracted and analysed with statistical software. Here, we present an R package to extract, visualize and generate otolith shape data with a small number of easy-to-use functions. There are built-in functions which allow users to perform automatic processes such as extract the otolith outlines from images, visualize the mean shape, smooth the outline by eliminating pixel noise [7] and transform the outlines into independent coefficients using either Normalized Elliptic Fourier or Discrete Wavelet, which can be entered into a wide range of statistical packages in R. The Wavelet transform provides a powerful alternative to the more commonly applied Fourier transform in shape analysis. While the Fourier transform provides functions in the form of sines and cosines which are non-local and can therefore result in poor approximations of sharp edges, the Wavelet transform uses approximating functions that are contained in finite domains making them well-suited for approximating sharp edges [8].

## Methods

The shapeR package is written in the programming language R [9]. The functions are listed in the S1 Table. The package uses commands from the R packages gplots [10], ipred [11], jpeg [12], pixmap [13] and wavethresh [14]. All R source code is publicly available via GitHub (see 'Availability' section).

### Images

**Fig 1:**
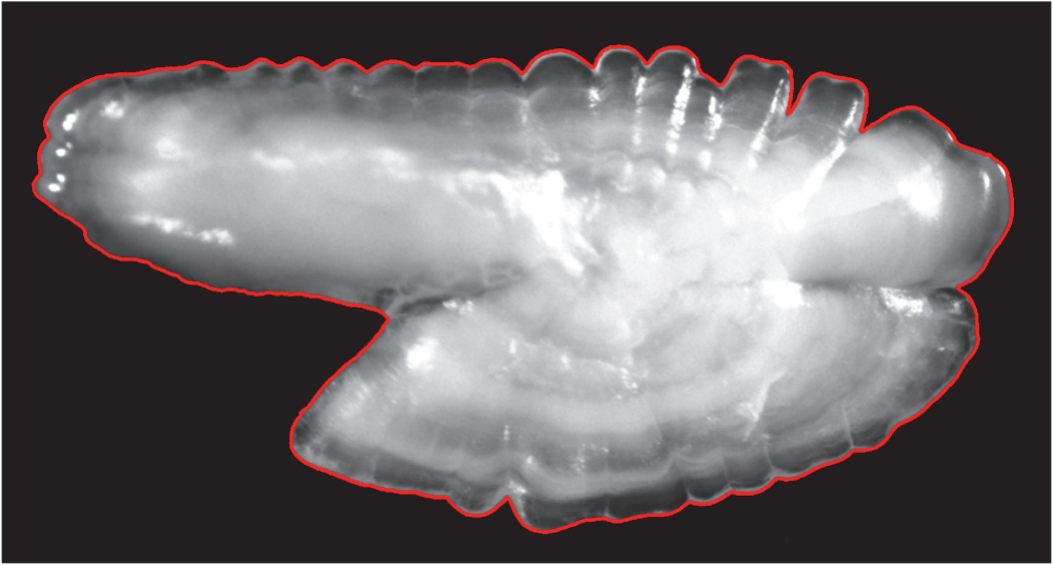
Example of an otolith image. The red outline marks the shape of the otolith which is extracted by shapeR and forms the basis for the analysis of variation within and among populations.

The first step of the shape analysis is to capture the otolith images (Fig. 1) using a dissection microscope with a digital camera attached. The microscope should be tuned so an otolith on a black background is as clear as possible. When the settings are ready, an image of a calibration measurement stick, in good focus, is taken as a size reference. Images should then be taken and stored in full color, ensuring good focus and high resolution in jpeg format (*.jpg). The otoliths need to be orientated with their rostrum to the left as seen in Fig. 1. For the ease of handling the images, make a folder called 'ShapeAnalysis' and store the images from each sampling unit in a unique area-folder within a folder called 'Original', and make a copy of the whole folder 'Original' and name it 'Fixed'. The folders 'Original' and 'Fixed' need to exist because images in both folders are used when the shapeR package is used to perform quality checks on the otolith outlines. The area-folders in the folders 'Original' and 'Fixed' should be named with two letters of the sampling unit, or country, and the station number of the sample. For example, 'IC' would represent a sample from Iceland. An otolith image name in folder 'IC' should be in the format '403_1', '403_2', '403_3', etc where the first three letters represent the station number and the second number, after the underscore, represents the fish number.

### Data files

A data file for each fish specimen (in rows), with information in columns such as population, station nr, sampling date, location, length, maturity stage, etc. (see data file example) is stored in the 'ShapeAnalysis' folder as a text file in a csv format (*.csv). Two columns in the data file are mandatory, 'folder' (consists of folder names such as 'IC') and 'picname' (consists of file names e.g. '403_1'), which are used to link biological information for each fish to the otolith outline. The column 'length_cm' needs to exist so it is possible to remove the allometric growth effect on otolith shape [15,16]. If other measurements are used (fish weight, otolith weight etc.) a column for each parameter needs to be given in the data file. Summary statistics of each otolith (otolith area, length, perimeter and width) can be obtained if the calibration measurements in pixels have been registered in the csv data file in a column labelled 'cal' (see example data file). To get the calibration measurements, use an image manipulation program and measure 1mm on the calibration measurement stick and register how many pixels 1mm is into the column 'cal'. When new images are placed into the area-folders in 'Original' and 'Fixed', the detect.outline function will detect them automatically. The image files are read into R using the functions readJPEG from the jpeg package [12] and pixmapGrey from the pixmap package [13].

### Sample Dataset

We present an example of otolith shape analysis on three discrete herring populations in the NE-Atlantic, from Iceland (n = 65), Norway (n = 65) and Scotland (n = 30). Example data set and images can be retrieved from GitHub (see 'Availability' section).

In R, load shapeR and retrieve the example data file with the commands library(shapeR) and data(FISH). To start the analysis, the project path needs to be set to the folder 'ShapeAnalysis' which contains the folders 'Original', 'Fixed' and the data file 'FISH.csv'. If the folder 'ShapeAnalysis' is on your Desktop, read in the data in the following way: > shape = shapeR("C:/Desktop/ShapeAnalysis", "FISH.csv")

### Outline extraction

To obtain the outline of each otolith we run the outline detection command detect.outline using the conte and regularradius functions [17]. The outlines are detected by first transforming the images into gray-scale. The images are then binarized using a threshold pixel value (intensity threshold) which can be defined by the user. The outlines are then collected automatically from all images in the folder 'Fixed'. Modification of the outlines are stored in different slots within the shape data object. Different comments will assess data in the different slots as referred to below:

> shape = detect.outline(shape, threshold = 0.2, write.outline.w.org = TRUE)

The threshold argument is used to distinguish the white otolith from the black background. The write.outline.w.org argument determines whether the detected outline should be written on top of the original image (TRUE) or not (FALSE) in the folder 'Original_with_outline' which shapeR makes automatically and places into the folder 'ShapeAnalysis'. It is good practice to run first 10 images and measure the time it takes to extract the outlines so the total run time can be estimated as it varies between computers and image resolution. Extracting each outline from the otolith images with the argument write.outline.w.org = TRUE takes ~5 seconds, while having the argument FALSE takes ~0.6 seconds using a computer with operating system Windows 8.1 and an Intel Core i5–3337U CPU 1.8 GHz Processor. It is recommended to run the images with the write.outline.w.org = TRUE the first time the images are run for quality checking, to see if the outline fits the original image from the microscope. If an error occurs, or the outline is of low quality, the outline can be removed from the shapeR instance:

> shape = remove.outline(shape,"IC","403_54")

Try to run again detect.outline with a different threshold e.g. with a higher threshold of 0.3. Try also mouse.click = TRUE which is added to the detect.outline command arguments and click on the center of the otolith. If that does not work, try to fix the image with an image manipulation program and get a better contrast between the otolith and the background in the 'Fixed' folder and run the detect.outline function again. It will only process again the otoliths which were removed and add them to the list of the other outlines.

It is possible to view one particular outline with:

> show.original.with.outline(shape,"IC","403_54")

### Contour smoothing

When the outlines have been captured from the images, the digitized outlines can have high frequency pixel noise around the outlines that can corrupt the Fourier or Wavelet analysis [7]. To eliminate pixel noise, it is possible to calculate a weighted moving average over three successive coordinate points using the function smoothout [17] to smooth multiple outlines. The number of iterations (n = 100 in the example) provided by the user is the maximum number of iterations of smoothing. The run time to smooth one outline takes ~0.03 seconds (see computer specifications in the "Outline extraction" section). To perform smoothing on the outlines:

> shape = smoothout(shape, n = 100)

otherwise omit this step.

### Shape coefficients

**Fig 2:**
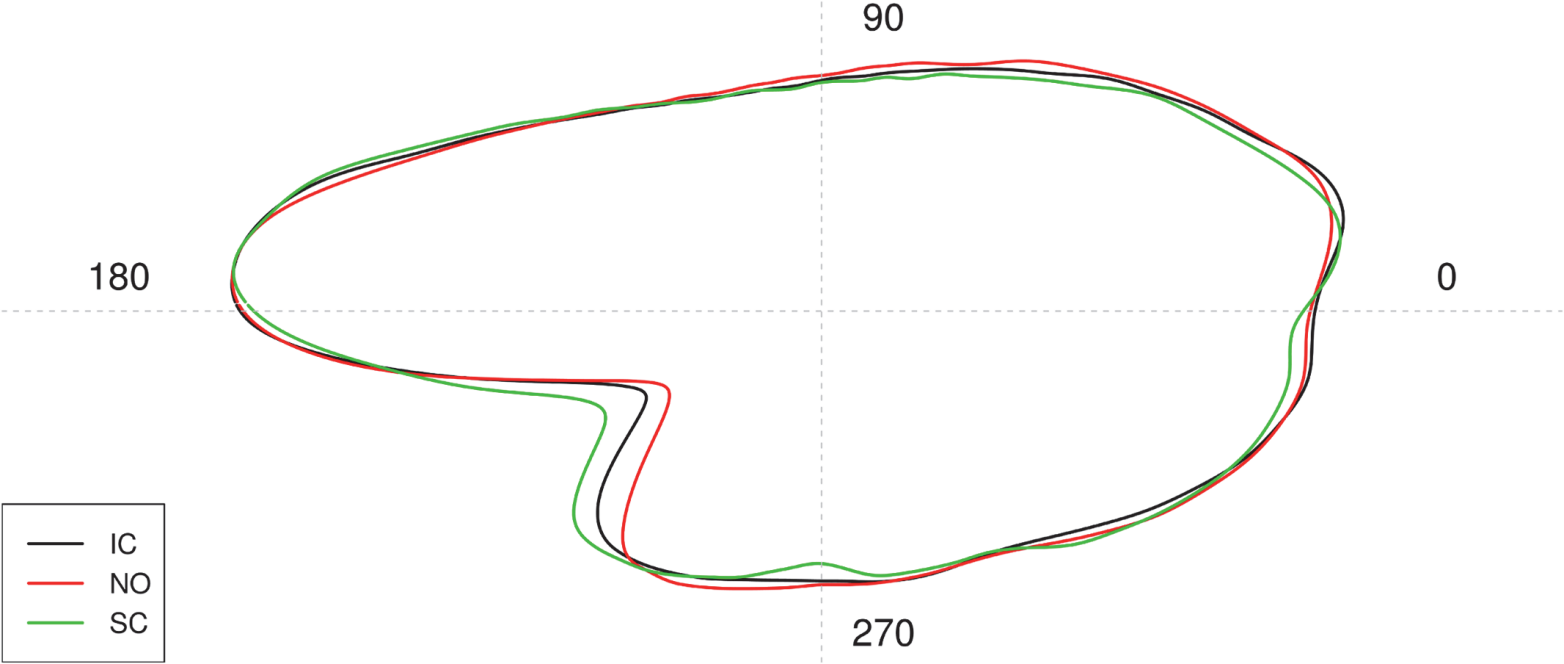
Mean otolith shape based on Wavelet reconstruction for three discrete fish populations from Iceland (IC, n = 65), Norway (NO, n = 65) and Scotland (SC, n = 30). Numbers represent angles in degrees (°) based on polar coordinates (see Fig. 4). The centroid of the otolith (center of the cross) is the center point of the polar coordinates.

When all the outlines have been captured with high quality, the shape coefficients can be extracted using the function generateShapeCoefficients. Before the Wavelet transformation, the rotation of all otoliths are positioned horizontally along the longest axis of the otoliths and the area is set equal in all (area = 1). Polar coordinates are then collected by drawing a polar axis (radial) horizontally from the otolith centroid (i.e. the mean of the x and y coordinates of the outline) to the right which corresponds to the 0° angle of the otolith outline (Fig. 2). From the 0° angle, radials are collected counter clockwise towards the 360° angle with equidistant angles between successive radials. The Wavelet coefficients are obtained using the functions wd and wr in the wavethresh package [14]. For Fourier, the Normalized Elliptic Fourier technique is performed using the iefourier and efourier functions [17] which both normalizes the otoliths with regards to size and rotation and collects the coefficients. Ten Wavelet levels give a total of 64 Wavelet coefficients using the Daubechies least-asymmetric Wavelet [18] and 12 harmonics give 45 Normalized Elliptic Fourier coefficients (48−3 = 45, the first three coefficients are omitted due to standardisation in relation to size, rotation and starting point). The coefficients are collected with:

> shape = generateShapeCoefficients(shape)

To connect the data file containing information on origin and size of the fish to the outlines, run:

> shape = enrich.master.list(shape)

It is recommended to save the shape object regularly:

> save(shape,file = "test.RData")

### Summary statistics

The maximum or Feret length and width of the otolith, its perimeter and area can all be collected with:

> getMeasurements(shape)

For each fish population ("pop"), the mean for the variables in the summary statistics (area, length, perimeter, width) can be calculated:

> tapply(getMeasurements(shape)$otolith.area, getMasterlist(shape)$pop, mean)

If the calibration measurements vary between figures, the area can be adjusted by the appropriate scale for each otolith.

### Mean otolith shape

The mean shape using the Wavelet coefficients is plotted in Fig. 2. To base the analyses on the Fourier coefficients instead of Wavelet, replace 'Wavelet' with 'Fourier' in all commands.

> plotWaveletShape(shape, "pop", show.angle = TRUE, lwd = 2,lty = 1)

### Adjusting coefficients for fish length

To adjust the otolith shape with respect to allometric relationships with the fish lengths [15,16], stdCoefs evaluates each Wavelet and Fourier coefficient. Those coefficients which show interaction (*P*<0.05), between population and length, are omitted automatically. In order to account for increased alpha error due to multiple testing of the different coefficients it is possible to conduct the Bonferroni adjustment [19].

> shape = stdCoefs(shape, classes = "pop", "length_cm", bonferroni = FALSE)

Using the Wavelet coefficients, three coefficients showed an interaction with fish length and were thus omitted, while applying the Bonferroni adjustment they were all included. The inclusion of these three coefficients did not affect the result of the overall analyses presented below.

### Reconstruction

The quality of the Wavelet and Fourier reconstruction can be estimated by comparing how it deviates from the otolith outline.

> est.list = estimate.outline.reconstruction(shape)

> outline.reconstruction.plot(est.list, max.num.harmonics = 15)

**Fig 3:**
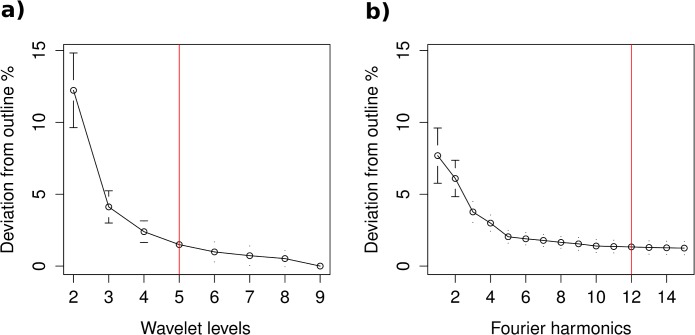
Quality of the a) Wavelet and b) Fourier outline reconstruction. The red vertical lines show the level of Wavelet and number of Fourier harmonics needed for a 98.5% accuracy of the reconstruction.

As seen in Fig. 3, the quality increases as expected with the number of Wavelet/Fourier coefficients used.

**Fig 4:**
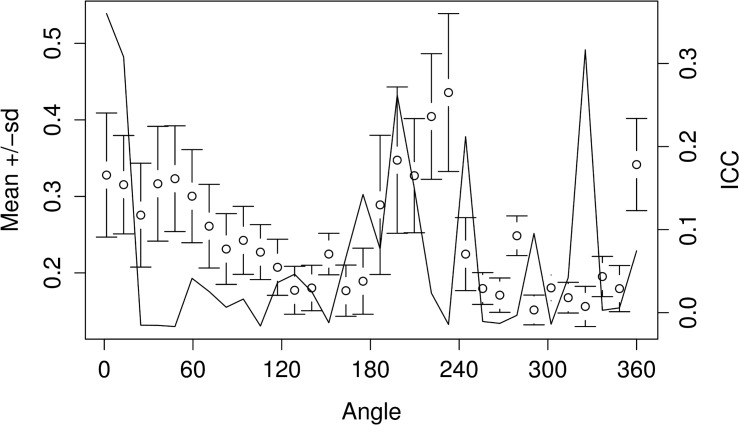
Mean and standard deviation (sd) of the Wavelet coefficients for all combined otoliths and the proportion of variance among groups or the intraclass correlation (ICC, black solid line). The horizontal axis shows angle in degrees (°) based on polar coordinates (see also Fig. 2) where the centroid of the otolith is the center point of the polar coordinates.

To inspect how the variation in the Wavelet coefficients is dependent on the position along the outline, the mean and standard deviation of the coefficients can be plotted against the angle (Fig. 4) using plotCI from the gplots package [10]. The proportion of variation among groups, the intraclass correlation (ICC), gives further information about the partition of the variation along the outline:

> plotWavelet(shape, level = 5, class.name = "pop", useStdcoef = TRUE)

Based on the patterns in Fig. 4, it is clear that most of the variation among groups can be traced to two areas of the otolith, angles 0–20° and 210–230° (see also Fig. 2) which correspond roughly to the postrostrum and the excisura major [20], respectively.

## Results

### Using shapeR output in other R packages

Output of the shapeR package can be analysed further using statistical methods implemented in R or other software. Here examples are presented on analyses of smoothed herring otoliths but to ensure a rigorous analysis the user should consider further the requirements of the statistical tests applied such as the number of predictor variables (relative to sample size), their multicollinearity and the independence of sampling units.

To analyse the variation in shape among the populations we apply Canonical Analysis of Principal Coordinates (CAP) [21] using the vegan package [22] on the length standardized Wavelet/Fourier coefficients with smoothed and unsmoothed outlines. The Wavelet coefficients can be analysed in the following way:

> library(vegan)

> cap.res = capscale(getStdWavelet(shape) ~ getMasterlist(shape)$pop)

Note the number of specimens needs to be larger than the number of coefficients.

The partition of variation among groups in the distance based on ANOVA can be tested using an ANOVA like permutation test (anova.cca), also in vegan [22] (see results in Table 1):

> anova(cap.res, by = "terms", step = 1000)

**Table 1:** Comparing otolith shape among three herring populations using an ANOVA like permutation test for smoothed and unsmoothed outlines.

Method	df	Var_unsm_	Var_sm_	*F*_unsm_	*F*_sm_	*P*
**Fourier**						
Model	2	0.17	0.18	14.83	18.30	0.001
Residual	157	0.88	0.76			
**Wavelet**						
Model	2	0.25	0.24	19.33	19.34	0.001
Residual	157	1.01	0.99			

Output from the R package shapeR, Fourier and Wavelet coefficients, were entered into the vegan package [22]. Differences among samples were tested by 1000 permutations. Df: degrees of freedom, Var: Variance among populations, *F*: pseudo *F*-value, *P*: proportion of permutations which gave as large or larger *F*-value than the observed one, for each test based on the smoothed and unsmoothed data.

#### Cluster analysis

**Fig 5:**
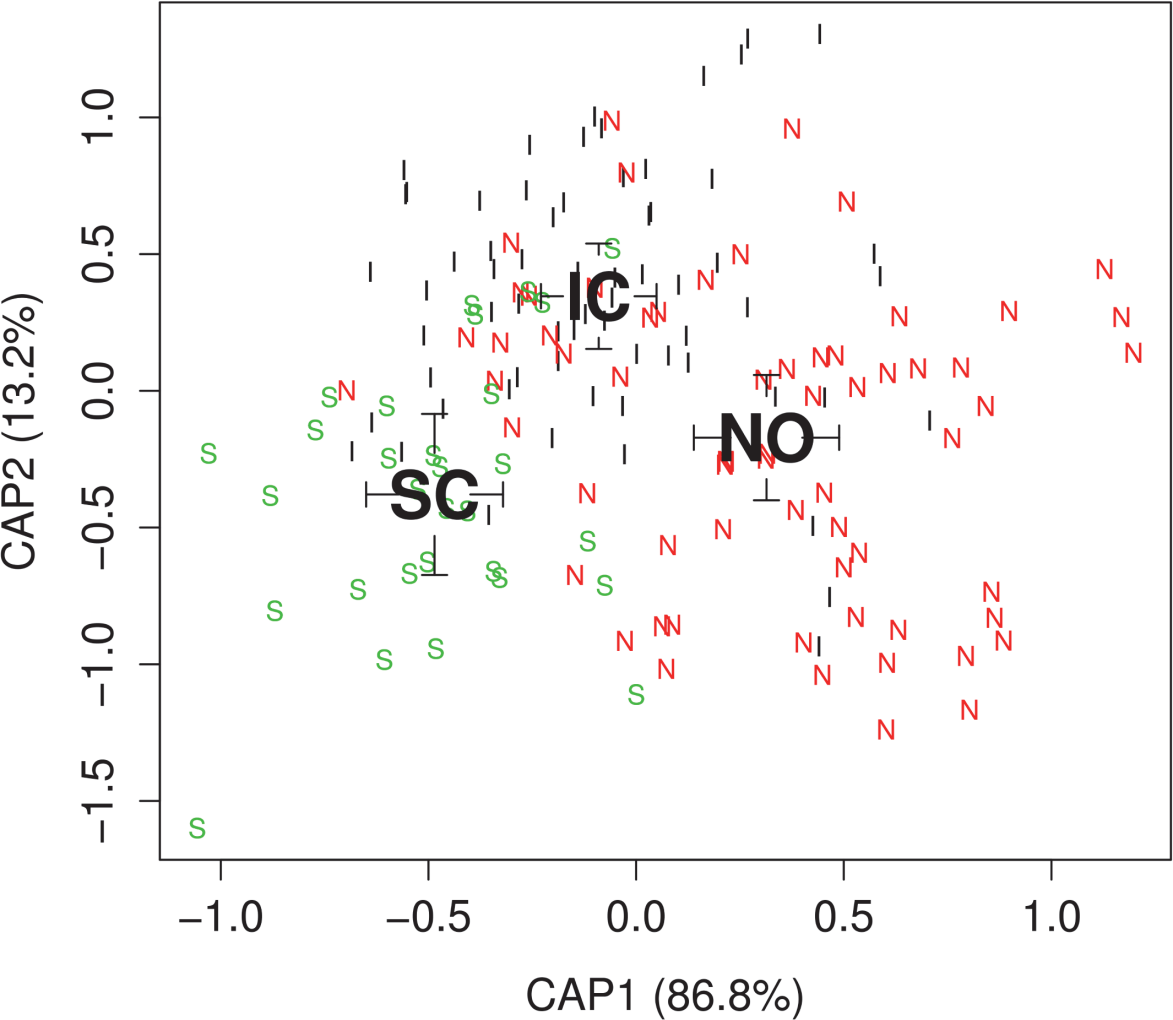
Otolith shape of samples from three herring populations in the NE-Atlantic using Canonical analysis of Principal Coordinates with the Wavelet coefficients. Canonical scores on the first two discriminating axes CAP1 and CAP2 are shown. Black letters represent the mean canonical value for each population, Iceland (IC), Norway (NO) and Scotland (SC) and smaller letters represent individual fish showing the first letter of each population. Interval surrounding the mean canonical values present one standard error (mean +/- 1SE).

For visualizing the clustering of the CAP results using the Wavelet coefficients in two dimensions (Fig. 5):

> eig = eigenvals(cap.res,constrained = T)

eig.ratio = eig/sum(eig)

cluster.plot(scores(cap.res)$sites[,1:2],getMasterlist(shape)$pop,

xlim = range(scores(cap.res)$sites[,1]),

ylim = range(scores(cap.res)$sites[,2]),

xlab = paste("CAP1 (",round(eig.ratio[1]*100,1),"%)",sep = ""),

ylab = paste("CAP2 (",round(eig.ratio[2]*100,1),"%)",sep = ""), plotCI = TRUE,conf.level = 0.95,las = 1)

The Canonical Analysis of Principal coordinates gives an overview of the differentiation in otolith shape among the three populations which were found significant by the ANOVA (Table 1, Fig. 5, *P* = 0.001). The Scotland sample differs from Norway and Iceland along the first discriminating axis (CAP1) and the Iceland sample shows mainly deviation from the Norwegian sample along the second axis (CAP2). Similar results were observed with separate analyses based on the Wavelet and the Fourier coefficients. Using the Wavelet coefficients, CAP1 explained 86.8% of the variation among populations and CAP2 13.2%. The corresponding values for Fourier were CAP1 89.4% and CAP2 10.6%.

#### Classification of individuals

To demonstrate classification of individuals to their sampling origin, based on the population variation at the two locations (Iceland and Norway), we apply Linear Discriminant Analysis on the standardized Wavelet coefficients. We start by setting a filter to select which samples (i.e. IC and NO) should be classified:> shape = setFilter(shape, getMasterlist(shape, useFilter = FALSE)$pop %in% c("IC","NO"))

> pop = factor(getMasterlist(shape)$pop)

Estimation of the classifiers success rate based on the Linear Discriminant Analysis can be done with bootstrap or cross-validation using the errorest function in the ipred package [11]. Here we show an example of how to run a cross-validation estimation using the cv estimator:

>library(ipred)

>mypredict.lda <- function(object, newdata)

>predict(object, newdata = newdata)$class

> stdw = getStdWavelet(shape)

> pop = factor(getMasterlist(shape)$pop)

> dd = data.frame(stdw = stdw,pop = pop)

>errorest(pop ~., data = dd, model = lda, estimator = "cv", predict = mypredict.lda,est.para = control.errorest(nboot = 1000))

The overall score rate of the classifier based on 65 Icelandic herring and 65 Norwegian herring was 79.2% using cross-validation estimation, but was slightly less using unbiased bootstrap (73.4%) and biased bootstrap (68.1%, sd = 0.002).

## Discussion

The shapeR package allows users to easily collect and analyse otolith shape data. Its output can be useful in any comparative study both at the population and species level and might be used in studies of variation on any two dimensional objects. The package allows users to analyse a large number of images in an automatic manner, without the need of selecting data points like in landmark or procrustes analyses, which might be prone to error and may suffer from the Pinocchio effect; where variation at a single landmark might be distributed incorrectly relative to other landmarks [23]. The ability to conduct both Fourier and Wavelet analysis in a single package and compare the results from the two methods is useful because of the variability in otolith shape among fish species. For Atlantic herring, the Fourier and Wavelet methods produced similar results in terms of overall comparison of shape, however the Wavelet method was useful for detecting shape differences at specific regions which could be located at a given angle on the otolith outline. Studying the variability of coefficients at a given angle of the outline is not possible with the Fourier method, because it only provides information about overall differences in otolith shape, not localized differences. Therefore, for some fish species, Wavelet might prove to be better at explaining shape differences, while for others, the Fourier method might be more powerful to distinguish populations. A further evaluation of the applicability of the two transformation methods, Fourier and Wavelet, in otolith shape analysis is warranted.

Otolith shape can be analyzed with standard statistical methods. Here we demonstrated the use of two multivariate methods. The classifier based on linear discriminant analyses gave a high overall score of correct classification when considering two population samples. However a higher score was obtained when samples from the two populations were compared including a larger number of geographic samples [24], and thus different estimates of the linear coefficients. Whether the classifier can be improved by other methods, such as the use of machine learning techniques [25], is a subject of further studies.

Future improvements of the shapeR package would include adding a fine resolution option when plotting the otolith outline, so users are able to zoom in and see the contour with all of its points on a pixel level and on this level see the effect of smoothing as well and adding more options to ease the accessibility of variables. Other scientists are also encouraged to validate and improve the software or send us suggestions for further additions.

## Availability

The R package shapeR is available with all source code and test data on GitHub (https://github.com/lisalibungan/shapeR) and will be available on the CRAN repository.

## Supporting Information

S1 Table Functions in the R package shapeR. (DOCX)
