# Supplementary material for: ShapeR: An R Package to Study Otolith Shape Variation among Fish Populations
Source: PLoS One. 2015 Mar 24;10(3):e0121102. doi: 10.1371/journal.pone.0121102 (PMC4372608; doi:10.1371/journal.pone.0121102)
Supplement: S1 Table — (DOCX) [file pone.0121102.s001.docx]

|  | |  |
| --- | --- | --- |
| **Function name** | **Description** |  |
| cluster.plot | Plot data clusters |  |
| detect.outline | Detect otolith outline |  |
| enrich.master.list | Link information in the info.file to the coefficients obtained from the otolith images |  |
| estimate.outline.reconstruction | Estimate the parameter reconstruction against the original images outlines |  |
| FISH | Example data file |  |
| generateShapeCoefficients | Get Wavelet, Fourier and basic shape variables |  |
| getFourier | Get Fourier coefficients, filtered according to filter |  |
| getMasterlist | Get filtered master.list value |  |
| getMeasurements | Get all otolith measurements (otolith area, length, perimeter, width) from unstandardized otoliths |  |
| getStdMeasurements | Get all otolith measurements (otolith area, length, perimeter, width) from standardized otoliths |  |
| getStdFourier | Get standardized Fourier coefficients, filtered according to filter |  |
| getStdWavelet | Get standardized Wavelet coefficients, filtered according to filter |  |
| getWavelet | Get Wavelet coefficients, filtered according to filter |  |
| outline.reconstruction.plot | Plot outline reconstruction |  |
| plotFourier | Mean and standard deviation of the Fourier coefficients |  |
| plotFourierShape | Mean otolith shape based on Fourier reconstruction |  |
| plotWavelet | Mean and standard deviation of the Wavelet coefficients |  |
| plotWaveletShape | Mean otolith shape based on Wavelet reconstruction |  |
| read.master.list | Read updated master list |  |
| remove.outline | Remove otolith outline |  |
| setFilter | Set a filter to analyze the shape data |  |
| shape | An example shapeR instance including 160 images. The shape coefficients have not been generated |  |
| shape.coef | An example shapeR instance including 160 images. The shape coefficients have been generated |  |
| show-method | Show a shapeR object |  |
| [show.original.with.outline](http://127.0.0.1:42194/help/library/shapeR/html/show.original.with.outline.html) | Show one image with outline |  |
| smoothout | Remove high frequency pixel noise around the otolith outline |  |
| stdCoefs | Standardize coefficients |  |
| write.image.with.outline | Write outlines on top of the original images for quality checking |  |
|  |  |  |
|  |  |  |
